# Pulp Nodules and Their Treatment

**Published:** 1883-03

**Authors:** H. L. Gilmour

**Affiliations:** PhiladelphiaA.


### ARTICLE VI.

### *Pulp Nodules and Their Treatment.*

BY H. L. GILMOUR, D. D. S., PHILADELPHIA.

In writing upon a subject which seems surrounded with so much obscurity, both as to cause and diagnosis, I feel to shrink more from the possible criticisms of superficial readers and thinkers, than a sense of the magnitude of what is before me. I propose making reference only to cases in practice which it has been my opportunity to treat, leaving microscopy and deeper scientific research for those less accustomed to the ache and cure contact of every-day practice, but infinitely better able to delve into pulp *minutiæ* and territories still unexplored. The process of pulp calcification has been known for many years, and yet our knowledge with regard to it still remains vague.

My reference, however, is to what is best known as pulp stones or nodules, existing in the pulp tissue, independent of connection with the walls of the pulp chamber. These nodules, according to my own observation, vary in size from grains as fine as sand—more easily felt by pressure between the finger and thumb than seen by the eye—to large irregular shaped nodes, entirely filling the pulp cavity, and conforming to its shape. The dislodgment of these bodies is frequently attained with considerable difficulty. I have found a spear-shaped point most effective in breaking up the mosaic, by simply insinuating the point between the nodules and giving it a twist, when they may be easily removed by a stout nerve hook.

In the year just closed I had several cases, all in upper molars, and with the exception of one case, in the teeth of a gentleman ranging from forty to forty-five years of age, of nervo-sanguine temperaments, grand physical constructions, looking as if sympathy could never be evoked from any source.

The exception mentioned was in the mouth of a lady, and the tooth the first one I can recall where the cavity of decay was deep enough to expose the pulp; this exposure occurred at the *mesio-buccal* horn, the cavity extending under the gum. I made an arsenical application, seeing her, by appointment, the following day to renew the application; at that time I exposed the pulp over the palatal canal, and there came in contact with as large a nodule as I have in my collection. It was easily removed, with two-thirds of the palatal pulp adhering to it; after picking out four nodules, the patient reminded me of removing five or six nodules three years ago from the corresponding tooth on the other side.

There being no necessity for another arsenical application, I dressed the canals with acetate of morphia paste, filling the outside cavity with pellets of cotton and sandarach varnish as is usual in pulp cavity of operations, and arranged for filling the tooth in a week.

Another class of teeth in which pulp nodules exist, are those without a sign of decay, erosion, or outside manifestation of trouble; neither soreness on percussion, thermal changes, or any of the usual diagnostic signs; but a sensitiveness peculiar to the scratch of the finger-nail on the neck of the tooth, not corresponding in severity to the same kind of scratch above or below the edge of enamel (as the case may be) in upper or lower cuspids and bicuspids; so that, in these cases, symptoms and comparative signs are our best and only guides in trying to arrive at a diagnosis, and just here is where caution, courage, and capability go hand in hand with the dental engine, and a sharp spear-pointed bit to gain access to the citadel of pain, and route the intruding foreigner. During the past year, instead of drilling into the crowns of this kind of teeth, making three, four or five arsenical applications to destroy the pulp, I drill a small hole at the edge of the gum or where judgment would direct, so as to enter on the *pulp side* of the nodules; and one application made in this way is as efficient for the purpose intended as five or six entering the crown ; the opening is easily stopped, with no possibility of displacement by attrition, until the next appointment for finishing, if possible, the operation.—*Dental Practitioner.*

---

### ARTICLE VII.

### *Plaster of Paris vs. Modeling Composition.*

